# Aluminium Nanoparticles as Efficient Adjuvants Compared to Their Microparticle Counterparts: Current Progress and Perspectives

**DOI:** 10.3390/ijms23094707

**Published:** 2022-04-24

**Authors:** Ali Nazarizadeh, Alexander H. Staudacher, Nicole L. Wittwer, Tyron Turnbull, Michael P. Brown, Ivan Kempson

**Affiliations:** 1 Future Industries Institute, University of South Australia, Adelaide, SA 5095, Australia; ali.nazarizadeh-khangheshlaghi@mymail.unisa.edu.au (A.N.); tyron.turnbull@unisa.edu.au (T.T.); 2 Translational Oncology Laboratory, Centre for Cancer Biology, SA Pathology and University of South Australia, Adelaide, SA 5000, Australia; alex.staudacher@sa.gov.au (A.H.S.); nicole.wittwer@sa.gov.au (N.L.W.); michaelp.brown@sa.gov.au (M.P.B.); 3 School of Medicine, University of Adelaide, Adelaide, SA 5000, Australia; 4 Cancer Clinical Trials Unit, Royal Adelaide Hospital, Adelaide, SA 5000, Australia

**Keywords:** aluminium, adjuvant, cancer, vaccine, nanotechnology

## Abstract

Aluminium (Al) compounds are used as adjuvants in human and veterinary prophylactic vaccines due to their improved tolerability compared to other adjuvants. These Al-based adjuvants form microparticles (MPs) of heterogeneous sizes ranging from ~0.5 to 10 µm and generally induce type 2 (Th2)-biased immune responses. However, recent literature indicates that moving from micron dimension particles toward the nanoscale can modify the adjuvanticity of Al towards type 1 (Th1) responses, which can potentially be exploited for the development of vaccines for which Th1 immunity is crucial. Specifically, in the context of cancer treatments, Al nanoparticles (Al-NPs) can induce a more balanced (Th1/Th2), robust, and durable immune response associated with an increased number of cytotoxic T cells compared to Al-MPs, which are more favourable for stimulating an oncolytic response. In this review, we compare the adjuvant properties of Al-NPs to those of Al-MPs in the context of infectious disease vaccines and cancer immunotherapy and provide perspectives for future research.

## 1. Introduction

Adjuvants have been defined as “agents that act non-specifically to increase the specific immune response or responses to an antigen” [1]. In other words, if an antigen only induces weak immunostimulation, an adjuvant can enhance the immune response to this antigen. The immunogenicity of vaccines, especially for the majority of subunit vaccines, often relies on this effect [2]. Among various clinically approved adjuvants, aluminium (Al) salts are the oldest and the most well-established. In 1926, Glenny proposed Al salts as adjuvants, which is a practice that has continued in human and veterinary prophylactic vaccines because, compared to other adjuvants, they are safer, better tolerated, and non-pyrogenic [3,4]. Surprisingly, after nearly a century of use, the precise mechanisms by which Al salts induce immunogenicity remain to be elucidated [5].

Despite the development of several adjuvants for prophylactic vaccines to fight against infectious diseases, the progress in therapeutic cancer vaccines has been slow, with only one FDA-approved vaccine (Sipuleucel-T; PROVENGE^®^) to treat metastatic castration-resistant prostate cancer in a limited group of nearly asymptomatic patients [6]. This slow progress can be attributed to two reasons: first, the available clinically approved adjuvants are not effective enough to promote migration of cytotoxic T cells (CTLs) to the tumour to induce oncolytic immune responses, and secondly, the compromised immune system of cancer patients impedes the induction of effective immune responses [7]. Furthermore, cancer cells are often protected against the host’s immune system by establishing an immunosuppressive tumour microenvironment (TME) characterized not only by ‘exhaustion’ of T-cell and natural killer cell responses but also accumulation of T-regulatory cells and other immunosuppressive phenotypes [8].

Recently, however, new opportunities for vaccine research have emerged through the identification of a strong size dependence on adjuvant material. Substantially different responses arise from nanoscale adjuvants. It has been observed that adjuvants at the nanoscale can initiate a remarkably robust and durable immune response [9,10]. Of significant importance, inorganic nanoparticles (NPs) such as gold, Al, and iron have unique physicochemical properties that enable them to be employed as efficient adjuvants. Their small size facilitates more efficient accumulation in lymph nodes and tumours [9]. Additionally, because of a larger surface area-to-volume ratio compared to their microparticle (MP) counterparts, greater amounts of other adjuvants such as CpG can be loaded on their surface, leading to stronger immune responses, which in turn can help overcome the important obstacles to cancer vaccine development [8,11].

**Figure 1:**
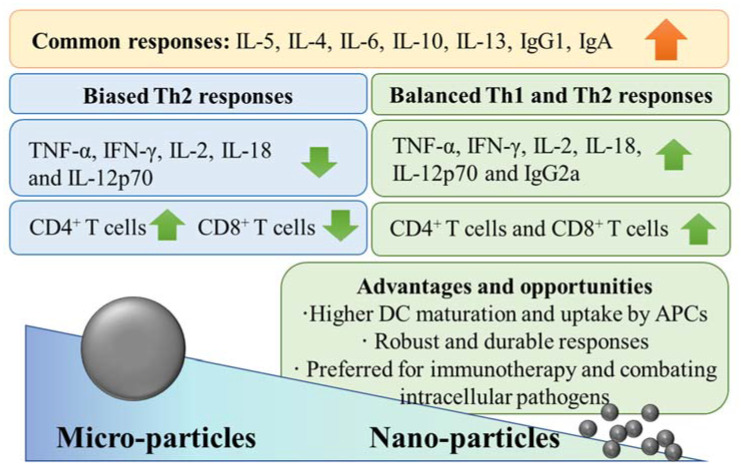
Generalized adjuvant effects of Al-MPs compared to Al-NPs.

Prospects for nanoadjuvants formulated in anticancer vaccines are emerging [9,10,12]. Several recent preclinical studies demonstrate that Al-NPs have numerous advantages over their MP counterparts, which make them a potential adjuvant for developing vaccines for intractable infectious diseases, such as tuberculosis, or as cancer immunotherapy, as depicted in Figure 1 [13,14,15,16,17]. Therefore, this review is focused on comparing adjuvant properties of Al-NPs to those of Al-MPs, and we discuss how nanoscale forms of already licenced Al adjuvants (hereafter referred to as microadjuvants) can dramatically change their activity. This has implications for how Al-NPs can be applied in cancer immunotherapies, which will also be discussed.

## 2. Al-NPs Show Different Adjuvant Properties Than Al-MPs

All commercial Al-based adjuvants share two common features: (1) They form MPs of heterogeneous size, ranging from ~0.5 to 10 μm, regardless of their chemical form (AlOOH or AlPO_4_,) [18]. (2) They bias humoral immunity toward type 2 responses (Th2) rather than type 1 responses (Th1), independent of their structure (crystalline or amorphous). The latter point means Al-MPs are ineffective for the induction of protective antigen-specific cell-mediated immune responses [19]. To be specific, the microadjuvants selectively induce a Th2 immune response in mice and a mixed response in humans, leading to activation of CD8^+^ T cells, which do not differentiate to CTLs. Therefore, Al-MPs are not preferred as adjuvants for vaccines against intracellular pathogens because cellular responses are usually required in these circumstances [20,21]. For example, Bungener et al. investigated the effect of Al microadjuvants on the magnitude and type of immune response induced by whole-inactivated virus (WIV) vaccine [22]. They immunized BALB/c mice once with a range of antigen doses of WIV produced from A/PR/8 influenza virus, either alone or in combination with the Al-MPs, and observed that the latter cohort developed Th2-baised responses evidenced by high IgG1 levels and a low number of IFNγ-producing T cells. Consistent with this, the animals vaccinated with a combination of WIV and Al microadjuvant suffered from more severe weight loss and had significantly higher viral loads in their lungs than the mice receiving WIV alone [22]. In agreement with these results, the available clinical evidence also supports the induction of humoral responses by microadjuvants [23].

Within the past couple of decades, researchers have discovered that Al-NPs can induce Th1 immunity, which is desirable for combatting intracellular pathogens [24]. In the late 1990s, Frey and colleagues proposed, for the first time, that Al oxide (Al_2_O_3_) NPs can be employed as an adjuvant for human immunodeficiency virus type 1 (HIV-1) [25,26]. They reported the synthesis of a conjugate composed of the C4 peptomer of HIV-1_MN_ gp 120 covalently linked to calcinated Al_2_O_3_-NPs with sizes ranging from 113 to 355 nm [25]. Mice immunized with the nanoadjuvanted vaccine produced robust antibody and T-cell responses against the C4 domain [26]. In 2008, Tang et al. compared the adjuvant effects of a conventional MP formulation of Al hydroxide (Al (OH)_3_) with a those of a nanosized counterpart loaded with the Newcastle disease antigen in chickens and observed that the nanoadjuvant initiated stronger Th1 and Th2 immune responses than the microadjuvant, which was evidenced by a marked increase in hemagglutination inhibition and antibody titre, as well as increased CD4^+^ and CD8^+^ mRNA expression in peripheral blood lymphocytes [27]. Subsequently, researchers conducted further comparative studies using different antigens to evaluate the possibility of exploiting Al-NPs as novel and efficient adjuvants for vaccines; this research is summarized in Table 1. A broad conclusion from this analysis of the literature is that Al-NPs induce more robust, durable, and balanced (Th1/Th2) immunity against pathogens than Al-MPs where cellular responses are required.

## 3. Reformulating Conventional Aluminium Microadjuvants into Nanoadjuvants

Alhydrogel^®^ (InvivoGen, San Diego, CA, USA) is an FDA-approved adjuvant containing Al as crystalline AlOOH-MPs, which can be temporarily dispersed by sonication or other high shear methods into nanosized particles; however, they can reaggregate quickly over a matter of hours (<10 h) [17]. To stabilise these nanoparticles, Orr et al. extracted well-dispersed rod-shaped Al(OH)_3_-NPs (~60 nm) from Alhydrogel^®^ by applying high shear force using a microfluidizer and stabilized them using an anionic polymer, polyacrylic acid (PAA) [17]. The resulting particles promoted Th1 immunity (Table 1). To test whether the effect was a generic property of the fabricated adjuvant, they replaced the negatively charged PAA with neutral polyethylene glycol (PEG) and identified that the PEG-nano-Alhydrogel^®^ did not induce significant Th1 responses. They concluded that induction of Th1 immunity was unique to PAA-nano-Alhydrogel^®^ [17].

High-molecular-weight (>300 kDa) crosslinked PAA polymers can potentially stimulate induction of Th1 responses and are the basis of the veterinary adjuvant Carbopol, which is formulated based on 450 kDa cross-linked PAA [39]. However, 2 kDa PAA (which was used to produce the PAA-nano-Alhydrogel^®^) did not promote Th1 immunity, suggesting that this adjuvant activity is specific to the PAA-nano-Alhydrogel^®^ compound and not only due to the presence of PAA. Moreover, an admixture of PAA and Alhydrogel^®^ did not provide adjuvant properties, indicating that the NP formulation was crucial for the observed activity [17].

Subsequently, it was demonstrated that the Th1 immunity against the recombinant tuberculosis antigen ID93 was structure-dependent and that PAA adsorption to Alhydrogel^®^ was a key parameter affecting the adjuvanticity of nano-Alhydrogel^®^. Various factors, such as the molecular weight of PAA and the formulation pH, influence the adsorption rate of PAA to the nano-Alhydrogel^®^ surface. Mice immunized with nano-Alhydrogel^®^-PAA formulated at pH 5.6 or lower and adsorbed with ID93 had a higher level of antigen-specific IgG and multifunctional CD4^+^ T cells isolated from splenocytes than mice vaccinated with adjuvants formulated at a pH of 7 and 7.6. This is most likely because the carboxylic acid groups in PAA are mostly protonated in an acidic pH range (<5.6), and therefore, adsorption to Alhydrogel^®^ is relatively high. Altogether, these findings suggest nanoadjuvants stabilized with surface-adsorbed polymers (PAA or PEG) have tuneable properties that affect immune responses [40].

Adju-Phos^®^ (InvivoGen, USA) is another FDA-approved adjuvant containing Al as amorphous and plate-like heterogeneously sized aggregates of AlPO_4_ up to several micrometres in diameter (50 nm–3 µm). Like Alhydrogel^®^ (and other Al adjuvants), Adju-Phos^®^ induces Th2-biased responses, but it has two main differences. First, Adju-Phos^®^ has a negative electrical charge at pH 5–7, enabling positively charged antigens to be adsorbed to it. At neutral pH, Alhydrogel^®^ is positively charged and thus readily adsorbs negatively charged antigens [20]. Second, Adju-Phos^®^ dissolves more readily at the injection site compared to Alhydrogel^®^, as demonstrated by intramuscular delivery in rabbits [20]. To improve the adjuvanticity of Adju-Phos^®^, Vrieling et al. prepared AlPO_4_-NPs by sonicating the commercial adjuvant. The prepared NP (~200–400 nm) was only stable for 14 days and had adjuvanticity similar to that of the microadjuvant (~1500 nm) when diphtheria toxoid was adsorbed on it [41]. To prevent reaggregation, the NP was stabilized with various amino acids, which resulted in increasing size (400–600 nm). Among the stabilizers, arginine significantly increased diphtheria toxoid-specific antibody titres compared to the naked NP but had no obvious effect on toxin-neutralising antibody titres [41]. It appears that not only reducing the size but also changing the Al-to-phosphate ratio can influence adjuvant properties of Adju-Phos^®^. To this end, Liang et al. engineered a library of amorphous Al-hydroxyphosphate-NPs with defined surface properties [42]. They identified that the positively charged NP (~21 nm) in which the Al ratio was higher than phosphate had better adjuvanticity than the negatively charged counterpart (~23 nm), where the phosphate ratio was higher than Al, as evidenced by the induction of higher levels of antigen-specific IgG and superior protection against *S. aureus* challenge. Additionally, none of the NPs stimulated ex vivo production of IFN-γ by splenocytes from mice vaccinated with human papillomavirus type 18 virus-like particles adsorbed on the NPs [42]. Based on this study and others in Table 1 [29], it appears that AlPO_4_-NPs cannot induce robust Th1 responses; however, this needs to be investigated in future studies.

## 4. Mechanisms of Al-NP Adjuvanticity

Just like their MP counterparts, it is not clear how Al-NPs induce immunogenicity. Simón-Vázquez et al. demonstrated in vitro that Al_2_O_3_-NPs (13.56 ± 8.37 nm) can activate the immune system and induce Jurkat cell proliferation by upregulation of IL-2 (T-cell clonal expansion), FASN (involved in fatty acid synthesis), BCL2A1 and NAIP (antiapoptotic), and MDM2 (inhibitor of p53) genes [43]. It also remains unclear how moving from micron dimensions towards the nanoscale can enhance adjuvant effects of certain Al compounds. To understand this, Xu et al. incubated differentiated THP-1 cells in the presence of lipopolysaccharide at 37 °C or 4 °C for 3 h with AlOOH NPs (<100 nm) or MPs (median diameter: ~4.87 μm) [44]. Then, the macrophages were collected after cold washing in PBS thrice and lysed, and the Al level was measured as relative Al^3+^ normalized to total protein content using inductively coupled plasma optical emission spectrometry. The results revealed that the Al obtained from the macrophages incubated with AlOOH NPs at 37 °C was significantly higher than that obtained at 4 °C (i.e., 87% higher). However, the level of Al obtained from the macrophages treated with the AlOOH MPs at 37 °C was not significantly different from that obtained at 4 °C. The authors attributed this lack of significant difference to the larger particle size of the AlOOH MPs and thus indirectly showed that the internalization rate of the AlOOH NPs (in terms of total Al) is higher than that of the AlOOH MPs, which was concluded as an underlying mechanism for stronger adjuvanticity of the NP [44]. It should be noted that washing Al adjuvant-incubated cells with PBS does not necessarily remove Al attached to extracellular surfaces [45]. Although Mold et al., using lumogallion as a fluorescence probe and transmission electron microscopy, directly visualized that undifferentiated and unprimed THP-1 cells can internalize Alhydrogel^®^, Adju-Phos^®^, and Imject^®^ in microparticulate form with sizes (outer diameter) around 0.9, 1 and 1.2 μm, respectively [46], there is no precise comparative study to clarify whether any difference in the adjuvanticity of Al-MPs and Al-NPs is due to differences in their cellular uptake by APCs. It is also unclear whether macrophage activation state plays any role in the particle internalization rate. It appears that following internalization, the above-mentioned AlOOH MPs and AlOOH NPs can activate the NLRP3 inflammasome pathway, resulting in secretion of the proinflammatory cytokine IL-1β by wild type and, to a lower degree, by NLRP3-deficient cells, with the NP having a more potent effect [28].

One of the most important components of innate immunity is a multiprotein complex known as the inflammasome, which employs pro-caspase-1 to process maturation of IL-1β and IL-18 cytokines by cleaving their precursors, pro-IL-1β and pro-IL-18. It also promotes pyroptosis, an inflammatory form of cell death [47]. The NLRP3 inflammasome has been reported to be involved in several diseases, including Alzheimer’s disease, prion diseases, type 2 diabetes, some infectious diseases, and cancer [48,49]. The precise mechanism of NLRP3 inflammasome activation is yet to be elucidated, but danger-associated molecular patterns (DAMPs), such as uric acid crystals, can potentially activate it [48]. Providing some insight, Thakkar et al. prepared AlOOH NPs (~30–100 nm) and MPs (~9.43 µm) adsorbed with OVA, intraperitoneally injected the formulations into BALB/c mice, and observed that the nanosized formulation led to a significant increase in uric acid levels in the peritoneal lavage, whereas the microsized formulation had no significant effects on these levels [50]. Furthermore, incubation of mouse J774A.1 macrophages with the NP increased uric acid levels in the culture medium, but the MP had again no significant effect [50]. Based on the above-mentioned study, it appears that a stronger adjuvant activity of Al-NPs is attributable to their higher capacity to induce DAMPs, such as uric acid production, which in turn activates NLRP3 inflammasome. Consistent results were also reported by Lebre et al., who observed that chitosan(CH)-Al NPs promoted NLRP3 inflammasome activation, which enhanced IL-1β secretion and induced DC maturation, as evidenced by increased surface expression of CD80, CD86, and CD40 [24]. Additionally, Orr et al. discovered that ASC (the inflammasome assembly protein), NLRP3, and IL-18R were all required for induction of Th1 response by Al(OH)_3_-NPs stabilized with PAA, highlighting the importance of inflammasome [17].

The activation of NLRP3 seems to be correlated with physicochemical properties of Al-NPs. To test this hypothesis, Sun et al. engineered a library of AlOOH nanorods with defined shape, crystallinity, and hydroxyl content [51]. They discovered that the secretion of IL-1β by THP-1 cells was not equal across the library. The largest nanorod, with a hydrodynamic size of 810 ± 67 nm, in water induced the highest secretion of IL-1β compared to any other species and commercial microadjuvant used at the same concentration and incubation time. Interestingly, the nanorod also had the highest uptake rate by differentiated THP-1 cells, signifying that higher internalization leads to more potent activation of the inflammasome. Following intraperitoneal injection of C57BL/6 mice with this nanorod, OVA-specific IgG1 and IgE titres were higher than in animals immunized with Al-MP-OVA [51]. In summary, the adjuvanticity of Al-NPs appears to be correlated with their uptake by APCs, which is governed by size, shape, and surface chemistry. Upon internalization, they activate the NLRP3 inflammasome pathway by inducing DAMPs, leading to secretion of proinflammatory cytokines.

## 5. Antitumour Immune Responses to Al-NPs

Considering that Al-NPs show different adjuvant properties than Al-MPs, as reviewed above in the context of infectious disease, their unique adjuvanticity has recently motivated researchers to exploit Al-NPs as novel and promising adjuvants for the induction of antitumour immune responses. Accordingly, the preclinical papers published within the past decade demonstrate that Al-NPs have been employed either as a component of cancer vaccines or (photo)immunotherapy (summarized in Table 2). Given that most tumour antigens are self-antigens, adjuvants for anticancer immunotherapy must be sufficiently potent to overcome the immune tolerance that may otherwise be induced by immunization with these antigens [52]. Sun et al. compared adjuvant effects of Al_2_O_3_-NPs (20–30 nm) to those of commercial Al(OH)_3_-MPs in BALB/c mice bearing H22 liver tumours. Mice were immunized with two subcutaneous injections of a tumour cell vaccine adjuvanted with either the NP or the MP and observed that the volumes of tumours in NP-immunized mice were smaller compared to mice receiving the conventional MP formulation [53]. Interestingly, the NP delayed tumour growth by increasing tumour infiltration of CTLs (assessed by a routine histopathology technique), demonstrating that Al-NPs contributed to antitumour immunity, whereas protective effects of the MP were weaker [53]. Li et al. evaluated the prophylactic effect of Al(OH)_3_-NPs (~112 nm) compared to its bulk counterpart (~9.3 µm) by immunizing C57BL/6 mice with OVA-adsorbed adjuvants on days 0, 7, and 14, followed by injection of syngeneic B16 melanoma cells expressing OVA on day 21 [15]. Compared to the mice receiving conventional adjuvant, where all mice (5/5) had palpable tumours, only one of the five mice immunized using the nanoformulation had a detectable tumour. Surprisingly, the antitumour response was reported to be antibody-mediated because OVA-specific CTL responses were not consistently detected [15]. In another study using a similar animal model, Yan et al. employed an Al-based nanomaterial with a similar chemical composition to that of Al-hydroxyphosphate-MP [54]. These Al-magnesium-layered double-hydroxide nanomaterials (40–200 nm) conjugated with CpG and OVA suppressed growth of murine melanoma tumours, induced OVA-specific antibodies, prolonged the survival of tumour-bearing mice, and increased tumour infiltration of CTLs. In contrast, the vaccine formulated with the MP did not elicit any potent antitumour immunity [54].

Despite the above-mentioned studies, which indicate Al-MPs either cannot or can only trigger weak antitumour immunity, Wang et al. discovered that in BALB/c mice with established H22 hepatocarcinoma, a protocol of repeated Al(OH)_3_-MPs (alum) injections induced an antitumour specific response that remarkably reduced tumour growth and prolonged survival [55]. The treatment consisted of the first alum dosing 5 days after cancer cell injection, with alum given every three or four days thereafter for a total of six injections. The treated mice were compared to a PBS-injected cohort, and toxicity assessments were limited to routine histopathologic examination of liver, kidney, and spleen without gross or microscopic examination of the injection site, with no signs of obvious toxicity reported [55]. However, further studies with more specific toxicity evaluations (e.g., organ-specific systemic biomarkers) are required to confirm that repeated injections of the MP can safely induce a robust antitumour immunity.

**Table 2:** Antitumour immune responses induced by Al-NPs.

Nanostructure	Study Mode	Principal Findings	Ref
OVA and two different adjuvants (CpG and 3pRNA †) were co-loaded on Al(OH)_3_-NPs (overall size: ~120 nm, diameter). The nanoformulation was used for vaccination.	DC2.4, Raw264.7, and BMDCs were used to assess uptake. BMDCs were used to assess antigen cross-presentation (measured as expression of H2-Kb-SIINFEKL complexes on BMDC surface). C57BL/6 was injected on days 0, 7, and 21 with the nanoformulation, and 7 days after the last injection, anti-OVA antibody levels were measured. C57BL/6 mice bearing B16-OVA tumours were used to assess anticancer effects of prophylactic intra-footpad vaccine injections given 26, 19, and 5 days before tumour inoculation or therapeutic intra-footpad vaccine injection 7 and 14 days after establishing the tumour.	The NP was internalized (~ 55–80%) by all 3 cell lines and enhanced cross-presentation of OVA. Additionally, it increased anti-OVA IgG levels. Whereas the formulation containing both 3pRNA and CpG induced the strongest IgG2a response, the formulation containing only 3pRNA induced the strongest IgG1 response. Vaccination also increased IFN-γ secretion in the spleen. Consistently, the population of IL-4+ CD4^+^ T cells and IFN-γ + CD8^+^ T cells were abundant in the spleen. Both prophylactic and therapeutic vaccines delayed tumour growth and prolonged mouse survival.	[56]
AlPO_4_ NPs (~50 nm) loaded with CpG, then coated with B16F10 cell membranes (overall size: ~60 nm). The nanoformulation was used for vaccination.	L929, DC2.4, and Raw264.7 cells were used to assess viability and uptake. BMDCs were used to assess maturation (measured as expression of CD80, CD86, and CCR7). C57BL/6 mice bearing B16F10 tumours were used to assess anticancer effects of prophylactic or therapeutic subcutaneous vaccine injections given 7, 14, and 21 days before tumour inoculation or after tumour establishment.	Cell viability was 90–100% (at concentrations up to 50 µg/mL) and >80% of DC2.4 and Raw264.7 cells for the NP formulation, which also induced maturation of BMDCs. Vaccines increased IFN-γ- and IL-4-expressing CD4^+^ T cells and IFN-γ-expressing CD8^+^ T cells in spleen and lymph nodes and concentrations of IL-6, IFN-γ, and TNF-α in culture supernatants of cell suspension from spleen or lymph nodes. The NP formulation induced mild skin inflammation at the injection site and no adverse histopathological effects in heart, liver, spleen, lung, or kidney. In contrast, mice injected with the MP had local skin inflammation and lymph node hyperplasia. Both prophylactic and therapeutic vaccines delayed tumour growth and prolonged mouse survival.	[13]
OVA and CpG were loaded on AlO(OH) NPs coated with polymer (PEG) (overall size: ~90 nm). The nanoformulation was used for vaccination.	Raw264.7, DC2.4, or BMDCs were used to assess uptake. BMDCs were used to assess lysosomal escape, antigen cross-presentation (measured as expression of H2-Kb-SIINFEKL complexes on BMDC surface), and DC maturation (measured as CD40, CD80, and CD86 expression, as well as TNF-α and IL-12p70 secretion). C57BL/6 mice bearing either B16-OVA or B16F10 tumours were used to assess biodistribution and anticancer effects of therapeutic intra-footpad vaccine injections given 7 and 14 days after tumour inoculation.	NP formulation was internalized (~100%) by all cell lines and enhanced cross-presentation of OVA. It was retained in draining lymph nodes, leading to an increase in the APC population and maturation compared to commercial microadjuvant. The NP (prime at day 0 and boosted at day 7) induced potent IgG1 and IgG2a responses, but the MP induced Th2-skewed immunity. IFN-γ + CD4^+^ cells and CTL populations, as well as TNF-α and IFN-γ levels, were higher in the culture (supernatants) of cell suspension from spleen isolated from NP-immunized mice compared to the MP-immunized cohort. The NP vaccine delayed tumour growth and prolonged mouse survival.	[16]
Rehydragel^®^ (Al(OH)_3_@heparanase, LV@HPA) was coated with polyethyleneimine (PEI) to synthesize LV@HPA/PEI nanoadjuvant. Then, OVA or tumour-derived autophagosomes (DRibbles) were adsorbed on the nanoadjuvant. The vaccine was formulated using LV@HPA/PEI-DRibble-DCs. The nanoformulation was used for vaccination ‡.	Murine DCs were used to assess viability and uptake. B3Z, BMDCs, and DCs were used to assess cross-presentation and DC maturation (measured as IL-12 secretion and CD80 and CD86 expression). C57BL/6 mice bearing PancO2 tumours were used to assess anticancer effects of therapeutic vaccine injections given subcutaneously 7 days and in the intra-inguinal lymph nodes 14 days after tumour inoculation. OT-1 mice were used to isolate splenocytes.	Cell viability was ~100% at concentrations up to 10 µg/mL. The NP promoted OVA uptake by the DCs (free OVA uptake: 7% vs. LV@HPA/PEI-OVA: 25.5%), DC maturation, and cross-presentation of OVA. The NP increased secretion of IFN-γ by CTLs isolated from tumour-bearing mice. No obvious body weight loss or abnormality were noticed in the immunized mice during the study course (54 days). Vaccination supressed tumour growth and prolonged mouse survival.	[57]
Aminophenol-functionalized α-Al_2_O_3_ NPs (~60 nm) conjugated with OVA aminophenol-functionalized α-Al_2_O_3_ NPs (~60 nm) conjugated with autophagosomes derived from 3LL cells. The nanoformulations were used for vaccination ^.	BMDCs were used to assess uptake. BMDCs, DC2.4, and OT-I T cells were used to assess cross-presentation (measured as expression of Kb-SIINFEKL on DC surface). Naïve C57BL/6 mice bearing B16–OVA tumours were used to assess anticancer effects of therapeutic vaccine injections given subcutaneously 7 days after tumour inoculation. C57BL/6 mice intravenously injected with 3LL lung tumour cells were used to assess anti-lung metastatic effects of therapeutic vaccine injections given subcutaneously 7 days after tumour inoculation.	NPs were internalized by DCs, which enhanced antigen cross-presentation and stimulation of naïve OVA-specific CD8^+^ T cells, leading to secretion of IFN-γ and IL-2. Animals immunized with NPs completely rejected tumours and remained tumour-free for >40 days, whereas the MP cohort succumbed to tumour burden. Subcutaneous injection of α-Al_2_O_3_-autophagosomes significantly suppressed lung metastases compared to the naked autophagosomes. The combination of vaccine and anti-OX40 antibody led to zero metastases in three of five mice, but no effect was observed in mice treated with anti-OX40 antibody alone.	[58]
Al sulphate and chlorin e6 (Ce6) were incorporated into bovine serum albumin (overall size: ~25 nm). The nanoformulation was used for photoimmunotherapy *.	B16F10 cells were used to assess uptake and photodynamic cytotoxicity. BMDCs were used to assess maturation (measured as expression of CD80, CD86, and CD40 and secretion of cytokines IL-6, IL-12/p70, and TNF-α). C57BL/6 mice bearing B16F10 tumours were used to assess biodistribution and anticancer effects of the nanoformulation intravenously injected 8 days after tumour inoculation.	NPs increased Ce6 uptake compared to free Ce6 without reducing cell viability (90–100%). NPs also enhanced cell-killing effects of irradiation and maturation of BMDCs. Following a single intravenous injection, NPs accumulated in tumours at 4-fold higher rate than free Ce6. Compared to locally injected commercial MPs, the NPs significantly reduced growth of primary tumours and metastatic foci and prolonged survival of the animals without causing substantial toxicity to other organs.	[14]

† 3pRNA: 5′-triphospate RNA. ‡ The nanoformulation size and physical properties were characterized, except the zeta potential. ^ In some mice, 100 mg anti-OX40 antibody was intraperitoneally co-injected with vaccine. * The animals were also subcutaneously injected around the tumour with four doses of CpG (3 μg per mouse per injection). Some of the findings appear to be due to CpG injection rather than the mere nanoformulation.

## 6. Concluding Remarks and Perspectives

For many years, Al salts were the only clinically licenced adjuvants in the U.S. As reviewed here, modifying already licenced Al microadjuvants to a nanoparticulate form can dramatically change their adjuvant properties and make them suitable for developing vaccines for which Th1 immunity is important, including infectious diseases such as tuberculosis, pertussis, and malaria or as cancer vaccines.

To date, no Al nanoadjuvant has been licenced for human use, although recent preclinical reports either in the context of infectious diseases (Table 1) or cancer research (Table 2) are very promising. Furthermore, the application of Al-NPs to induce oncolytic immune responses is emerging, and currently, only a limited number of preclinical papers is available. As summarized in Table 2, Al-NPs can promote antigen uptake by APCs and DC maturation, thus enabling more efficient antigen cross-presentation to naïve T cells and, ultimately, tumour infiltration of CTLs with fewer undesirable effects than Al-MPs at the injection site or at other organ sites. It is a well-established concept that an anaerobic and acidic TME impedes infiltration of immune cells, leading to chemo- and radioresistance and immune escape. An additional mechanism by which an oncolytic effect may be achieved is manipulation of the TME using Al NPs, which have been proposed as a means to neutralize extracellular acids [59,60]. The current evidence from in vitro studies suggests Al-NPs can potentially alkalinize culture medium [61,62]. Therefore, it appears that Al-NPs could not only prime the immune system for anticancer responses but also facilitate infiltration of CTLs by alkalinization of TME. However, this needs to be further validated in preclinical studies.

Considering that Al-NPs induce robust immune responses and potentially reshape TME, as described above, they may be exploited for chemo/radiosensitisation. In particular, inhibition of CTL infiltration into TME after radiotherapy has been claimed to be an important mechanism of treatment resistance [63]. Therefore, changes in the TME (e.g., immune cell populations) may enhance radiobiological response [64]. In this regard, it has been reported that Al-based nanostructures can promote anticancer effects of doxorubicin in vitro and in vivo [65,66]. Given that oncolytic immune responses induced by Al-NPs have mostly been assessed using mice bearing hepatocarcinoma or melanoma genetically modified to express the nominal antigen, chicken OVA, other cancer models should also be employed in future studies.

To date, proof of principle has been achieved with respect to consistent demonstration that nanoformulated Al adjuvants elicit immune responses distinct from those of formulations with larger particle sizes. Further research is required to develop a formulated product that exhibits an effective biological response, along with adequate stability/shelf-life without deterioration, dissolution, or molecular-level restructuring over time, which could affect the adjuvant properties and/or biocompatibility. Various stabilizing agents could be used to achieve this, but as discussed, may lead to diminution of the immune response through passivation of surface interactions. An up-scalable approach to preparation, along with characterization based on quality-by-design (QbD) principals to provide a consistent, reliable product will be beneficial for commercial production and regulatory approvals.

Although from a material science perspective, NPs have impressive properties, adverse biological effects may be associated with clinical toxicities [67]. Currently, there is no comprehensive research on toxicokinetics of Al-NPs in humans, and the available animal studies are generally limited to measuring the function of various organs using nonspecific biomarkers, biodistribution of Al-NPs, and routine histopathology examination following short-term exposure. Although there are many encouraging results that promote a benefit in using nanoformulated Al adjuvants, the long-term fate of Al-NPs needs to be elucidated in further detail, along with more thorough toxicity assessment, before these findings can be used to translate NP formulations to clinical trials.

## Figures and Tables

**Table 1:** Adjuvanticity of Al-NPs compared to microadjuvants and vaccines.

Adjuvant (Particle Size)	Vaccine Formulation	Principal Findings	Ref
Al(OH)_3_-NPs (~112 nm) vs. Al(OH)_3_-MPs (~9.3 μm)	Ovalbumin (OVA) and *Bacillus anthracis* protective antigen (PA) were adsorbed on the adjuvants.	OVA had more affinity to bind to the NPs than the MPs due to larger total surface area and more positive zeta potential of the NP. At equal OVA levels adsorbed on the particles, the NP induced higher anti-OVA IgG levels than the MP. The NP also induced higher anti-PA IgG levels than the MP 4 weeks after the second immunization. APCs internalized significantly higher levels of OVA adsorbed on the NP than the MP.	[15]
Al hydroxyphosphate NPs (<100 nm) vs. Al hydroxyphosphate MPs (~8–13 μm)	Egg lysozyme was adsorbed on the adjuvants.	The NP induced significantly higher antigen-specific IgG levels than the MP.	[28]
The sizes of Al(OH)_3_ (~0.99–1.96 μm) and AlPO_4_ (~1 μm) particles were reduced by applying high shear forces, then compared to Alhydrogel^®^ and a commercially available vaccine (TETAVAX).	Diphtheria toxoid was adsorbed on the adjuvants.	The size reduction improved protein loading capacity, boosted antidiphtheria antibody titration, and induced stronger Th2 antibody isotypes (IgG1 and IgA). Size-reduced Al(OH)_3_ adjuvant also induced stronger Th2 cytokines (IL-5, IL-6, IL-10 and IL-13).	[29]
Al(OH)_3_-NPs (~141.1 nm) vs. Bacillus Calmette–Guerin (BCG) vaccine	*Mycobacterium tuberculosis* ESAT-6-like protein EsxV was adsorbed on the Al(OH)_3_-NPs.	The NP stimulated secretion of Th1 cytokines, e.g., IFN-γ comparable to BCG.	[30]
Amorphous and crystalline forms of Al(OH)_3_-NPs (150–200 nm) vs. Alhydrogel^®^	*B. anthracis* protective antigen domain 4 (D4) was adsorbed on the adjuvants.	The NPs enhanced antigen uptake by THP-1 cells, induced more robust and durable Th1/Th2 responses evidenced by higher IgG1 and IgG2a levels compared to Alhydrogel^®^, and induced higher Th1 cytokine levels (IL-2 and IFN-γ). Conversely, Alhydrogel^®^ induced comparable or higher Th2 cytokine levels (IL-4 and IL-10). NPs prolonged survival of anthrax spore-challenged mice. The crystalline NP had moderate binding affinity compared to its amorphous counterpart, resulting in moderate antigen release (almost equal to Alhydrogel^®^).	[31]
Crystalline Al(OH)_3_-NPs (150–200 nm) vs. Alhydrogel^®^	D4 was encapsulated by non-ionic surfactant-based vesicles and adsorbed on the adjuvants.	The NP induced higher antigen-specific antibody titres (anti-D4 IgG) and IgG isotypes (IgG1 and IgG2a) than Alhydrogel^®^. It also stimulated splenocytes to produce both Th1 (IL-2, IFN-γ, and TNF-α) and Th2 (IL-4, IL-6, and IL-10) cytokines. The NP induced superior protection against anthrax spore challenge.	[32]
Al_2_O_3_-NPs (~30 nm) as a pulmonary vaccine adjuvant-delivery system vs. AlPO_4_-MP (2 μm)	OVA was adsorbed on the adjuvants.	NPs had significantly higher uptake by bone-marrow-derived dendritic cells (BMDCs) and promoted DC maturation to a higher degree, measured as CD40, CD80, and CD86 surface expression. NPs did not influence Raw264.7 (macrophage) cell viability at concentrations as high as 200 µg/mL. The NP induced more balanced Th1/Th2 responses, measured as anti-OVA IgG, mucosal IgA, and cytokine secretion (IFN-γ and IL-4), with only mild pulmonary inflammation.	[33]
Rod-shaped Al(OH)_3_-NPs stabilized with PAA (~60 nm) vs. Alhydrogel^®^	ID93 (*M. tuberculosis* vaccine antigen) or recombinant rHA (seasonal influenza hemagglutinin) were adsorbed on the adjuvants.	Unlike Alhydrogel^®^, the NP increased splenic IFN-γ-secreting CD4^+^ T cells and levels of Th1 cytokines, IL-18, and IL-12p70. The NP induced more robust and durable ID93-specific IgG1 and IgG2c antibodies, whereas Alhydrogel^®^ induced IgG1 antibody and was biased toward a Th2 response. The NP induced superior protection against lethal influenza challenge.	[17]
Al_2_O_3_-NPs (~28 nm), phospholipid bilayer-coated Al_2_O_3_-NPs (PLANs, ~33 nm) and the PEGylated PLANs (PEG-PLANs, ~31 nm) vs. AlPO_4_-MPs (~2 μm)	OVA was adsorbed on the adjuvants.	BMDC uptake of formulations ranked in the order of AlPO_4_-MPs<Al_2_O_3_-NPs<PEG- PLANs<PLANs. The microparticle reduced cell (Raw264.7) viability. NPs did not show cytotoxicity and promoted cell growth. NPs and, more specifically, PLANs promoted DC maturation, measured as CD40, CD80, and CD86 surface expression. PEG-PLANs accumulated in draining lymph nodes at significantly higher levels. Whereas PLANs and PEG-PLANs elicited stronger humoral responses than AlPO_4_-MPs, Al_2_O_3_-NPs did not. NPs induced Th1 responses (IgG2a> IgG1), and conversely, the MP induced Th2 responses. NPs increase IL-4 and IFN-γ levels, as well as CD8^+^ T cells, in spleen compared to the MP. PEG-PLANs were the most effective adjuvant.	[34]
AlOOH nanorods (ALNRs) functionalized with (3-aminopropyl) triethoxysilane (ALNR-NH_2_) or 3-(trihydroxysilyl)- 1-propanesulfonic acid (ALNR-SO_3_H) (diameter: 20 nm, length: 150–200 nm) vs. Imject^®^	OVA was adsorbed on the adjuvants.	THP-1 cell uptake of formulations ranked in the order of ALNR-NH_2_ = ALNR-SO_3_H< Imject^®^. Moreover, IL-1β secretion by THP-1 cells ranked in the order of ALNR-SO_3_H≤Imject^®^<ALNR-NH_2_. Cellular oxidative stress (measured as glutathione level) of formulations ranked in the order of ALNR-SO_3_H <alum<ALNR-NH_2_. ALNR-SO_3_H and Imject^®^ had the same capacity to induce anti-OVA IgG1 and IgE, whereas ALNR-NH_2_ induced significantly higher levels of the antibodies.	[35]
Amorphous AlOOH nanosticks (diameter: ~8 nm, length: ~80 nm) vs. Alhydrogel^®^ (~900 nm)	OVA was adsorbed on the adjuvants.	J774A.1 macrophage uptake of NPs was higher than that of Alhydrogel^®^. THP-1 cells treated with NPs released higher levels of IL-1β than Alhydrogel^®^. NPs induced higher levels of serum anti-OVA IgG and IgG1 than Alhydrogel^®^. Al nanosticks and Alhydrogel^®^ induced local subcutaneous nodule and granuloma formation, although the site injected with the Al nanosticks had a relatively lower cellularity.	[36]
Al(OH)_3_-NPs (~40 nm), phospholipid bilayer-coated Al(OH)_3_-NPs (PLAlOH_3_: ~50 nm) vs. Al(OH)_3_-MPs (~10 μm)	OVA was adsorbed on the adjuvants.	BMDC uptake of formulations ranked in the order of Al(OH)_3_-MPs<Al(OH)_3_-NPs<PLAl(OH)_3_. NPs induced more durable and higher anti-OVA IgG and IgA than the MP. PLAl(OH)_3_ induced balanced IgG2a>IgG1, contrary to the MP which induced biased Th2 response (very high level of IgG1). Whereas PLAl(OH)_3_ elevated both IL-4 and IFN-γ in serum and supernatant of splenocytes, the MP increased only the IL-4 level. The MP increased only CD4^+^ T-cell populations in the spleen, but the PLAl(OH)_3_ elevated both CD4^+^ T and CD8^+^ T-cell populations. The stimulation index for splenocyte proliferation was ~2-fold higher for PLAl(OH)_3_ than the MP. Following subcutaneous injection into a forelimb, PLAl(OH)_3_ was accumulated in axillary lymph nodes and taken up by DCs. Following intramuscular injection, neither NP induced local inflammation, but the MP induced severe inflammatory reactions.	[37]
Al_2_O_3_-nanowire (diameter: ~20–40 nm, length: ~20–60 µm) vs. Al_2_O_3_-MPs (20 µm scale) and Alhydrogel^®^ (2 µm)	OVA was adsorbed on the adjuvants.	All formulations were non-toxic to HeLa and THP-1 cells (up to 200 µg/mL); however, the nanowire was slightly toxic to U87MG cells (viability: ~70%) compared to Al_2_O_3_-MPs (viability: ~80%) and Alhydrogel^®^ (viability: ~85%) at the mentioned concentration. The nanowire induced higher levels of anti-OVA IgG than MPs 11 days after the second immunization. Cellular immune response, measured as delayed-type hypersensitivity, was stronger in nanowire-treated mice than the Al_2_O_3_-MP-injected cohort at 6–24 h after antigen exposure. Following injection into an air sac in the flank, the nanowire induced a lower degree of microvascular damage and oedema than Alhydrogel^®^.	[38]

## Data Availability

Data is contained within the article.
